# Onycho-pachydermo periostitis: Ixekizumab as a therapeutic option

**DOI:** 10.1016/j.jdcr.2025.06.066

**Published:** 2025-09-10

**Authors:** Lanyu Sun, Sónia Fernandes, Luís Soares-de-Almeida, Joana Antunes, João Janeiro, Paulo Filipe

**Affiliations:** a Serviço de Dermatologia, Unidade Local de Saúde Santa Maria, Lisboa, Portugal; b Clínica Universitária de Dermatologia, Faculdade de Medicina da Universidade de Lisboa, Lisboa, Portugal; c Serviço de Imagiologia, Unidade Local de Saúde Santa Maria, Lisboa, Portugal

**Keywords:** ixekizumab, nail psoriasis, onycho-pachydermo periostitis, psoriatic arthritis

## Introduction

Psoriatic onycho-pachydermo periostitis (POPP) is an uncommon form of psoriasis characterized by psoriatic nail abnormalities, connective tissue thickening, and periostitis of the distal phalanges, leading to a “drumstick” deformity.^1^^,^^2^ A diagnosis requires the presence of all 3 of these features. POPP can be highly painful and often leads to significant functional impairment.^1^ Treatment remains a challenge. We report the first documented case of POPP that demonstrated a partial clinical response to adalimumab, with further improvement following treatment with ixekizumab.

## Case report

**Fig 1:**
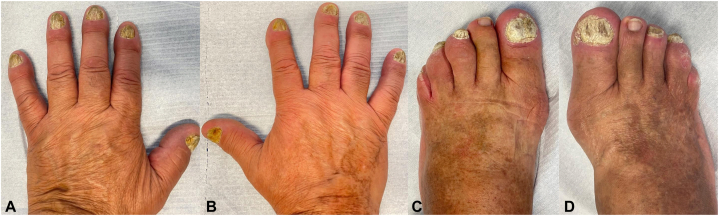
POPP: Clinical presentation with psoriatic onychodystrophy and drumstick-like swelling of almost all fingers (**A** and **B**) and toes (**C** and **D**), except for the second toe of both feet. *POPP*, Psoriatic onycho-pachydermo periostitis.

A 66-year-old man presented with a 1-year history of painful nail changes affecting both his fingers and toes. The condition was unresponsive to antifungal therapy. He had no personal history of dermatological conditions, and there was no family history of psoriasis or other skin diseases. On physical examination, he presented with tender, drumstick-like swelling of almost all fingers and toes, characterized by diffuse, fusiform enlargement of the digits, along with xanthonychia, subungual hyperkeratosis, and onycholysis (Fig 1, *A*-*D*).

**Fig 2:**
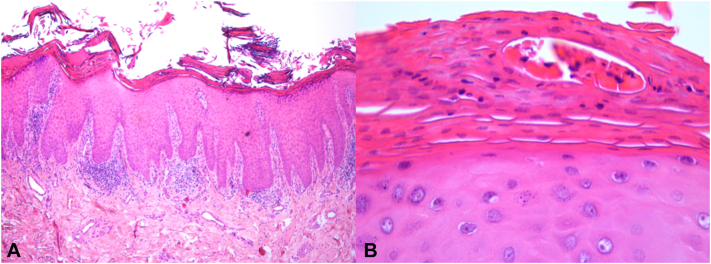
POPP: Histopathological features showing regular acanthosis of the epidermis, parakeratosis, hypogranulosis, and a lymphocytic infiltrate in the upper and mid-dermis (**A**); a Munro microabscess is observed in the stratum corneum (**B**) (hematoxylin and eosin staining).

No psoriatic lesions were found on the remaining skin. Laboratory tests, including the autoimmune panel, were within normal limits, except for a positive HLA-B27 antigen. Mycological examination of the nails was negative. A biopsy of a hyperkeratotic periungual lesion revealed regular epidermal acanthosis, areas of parakeratosis in the stratum corneum, Munro microabscesses, hypogranulosis, and a lymphocytic infiltrate in the upper and mid-dermis (Fig 2). These findings were consistent with psoriasis.

**Fig 3:**
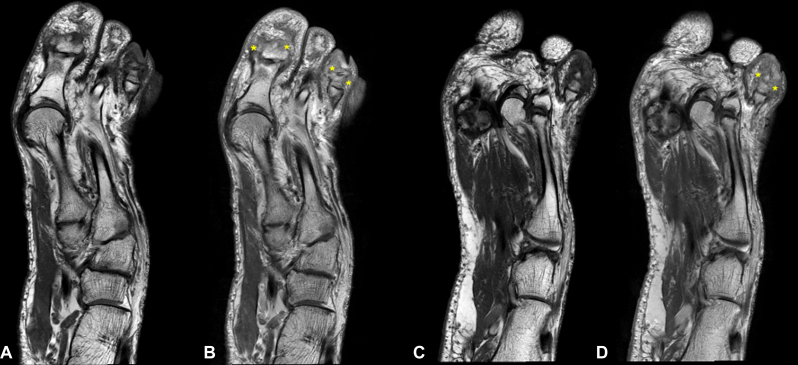
POPP: T1-weighted MRI of the left foot showing soft tissue edema with contrast enhancement postgadolinium (*asterisks*) in the first, third (**A** and **B**), and fourth toes (**C** and **D**). MRI: pre- (**A** and **C**) and post-gadolinium (**B** and **D**). *MRI*, Magnetic resonance imaging; *POPP*, psoriatic onycho-pachydermo periostitis.

Radiographs of the hands and feet showed no evidence of arthritis. T1-weighted magnetic resonance imaging (MRI) of the left foot revealed increased signal intensity and contrast enhancement in the first, third, and fourth toes (Fig 3, *A* and *B*). This hyperintensity was consistent with bone marrow edema in the affected phalanges, suggestive of periostitis.

**Fig 4:**
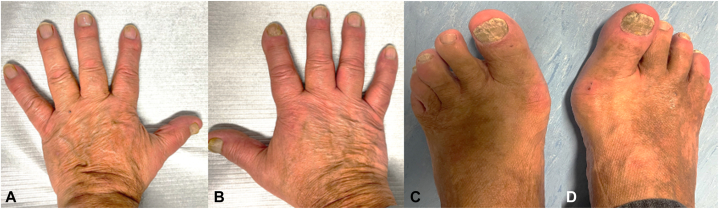
POPP: Clinical improvement of the fingers (**A** and **B**) and toes (**C** and **D**) after 1 year of treatment with ixecizumab. *POPP*, Psoriatic onycho-pachydermo periostitis.

**Fig 5:**
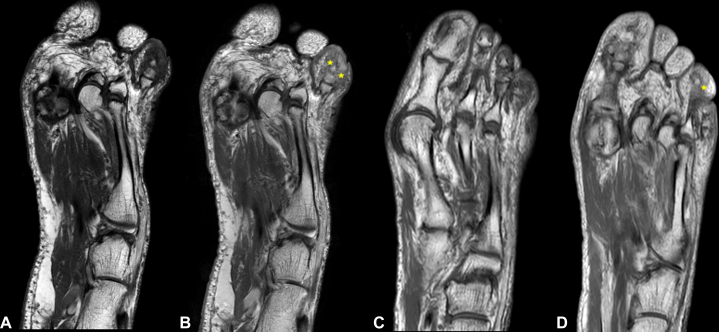
POPP: Follow-up T1-weighted MRI postgadolinium demonstrates decreased contrast enhancement and reduced soft tissue edema surrounding the fourth toe (*asterisks*), comparing baseline (**A** and **B**) and 1 year after treatment with ixekizumab (**C** and **D**). MRI: pre- (**A** and **C**) and post-gadolinium (**B** and **D**). *MRI*, Magnetic resonance imaging; *POPP*, psoriatic onycho-pachydermo periostitis.

Based on the clinical presentation and histopathological findings, a diagnosis of POPP was confirmed. The patient began treatment with adalimumab. After 1 year, some improvement was observed in the fingers; however, the response in the toes remained modest. As a result, therapy was switched to ixekizumab. This led to near-complete clearance of finger involvement and marked improvement in the toes after another year, both clinically (Fig 4, *A*-*D*) and on MRI (Fig 5, *A*-*D*). No adverse events were reported during treatment.

## Discussion

**Fig 6:**
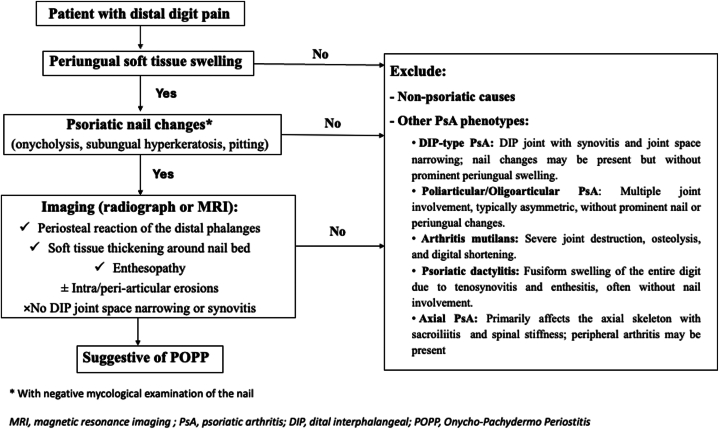
POPP: Algorithm for the differential diagnosis of POPP. *DIP*, Distal interphalangeal; *MRI*, magnetic resonance imaging; *POPP*, psoriatic onycho-pachydermo periostitis; *PsA*, psoriatic arthritis.

In 1989, Fournié *et al* introduced the term “POPP” to describe psoriatic onychosis associated with painful periungual soft tissue swelling and distal phalangeal periostitis, occurring in the absence of involvement of the adjacent distal interphalangeal (DIP) joint.^3^ The pathogenesis of POPP remains unknown. It is still unclear whether this condition represents a variant of DIP psoriatic arthritis (PsA) or a distinct form of PsA.^1^^,^^4^ Clinically, POPP patients present with pain, periungual swelling, and onychodystrophy, but without overt arthritis symptoms in the DIP joint. DIP joint arthritis typically involves joint pain, stiffness, and functional limitation centered on the DIP joint but often lacks the prominent soft tissue swelling and nail apparatus involvement seen in POPP.^2^^,^^5^ Given the lack of DIP joint involvement, the bony changes observed in POPP have been attributed to the anatomical relationship between the nail and the terminal phalanx; inflammatory processes may spread from the subungual dermis to the terminal phalanx through the fibrous septa.^1^^,^^5^ An association with HLA-B27 has been reported.^6^ Of note, the presence of neutrophilic infiltration and Munro microabscesses in POPP lesions suggests that neutrophils play a central role in its pathophysiology.^7^ The prevalence of this condition remains poorly documented in the literature. A 2021 review identified only 31 reported cases of POPP since 2000.^1^ The reported patients were predominantly male, with most over the age of 30 and a mean age of 44.9 years. Pediatric cases have also been documented.^8^ The duration of symptoms varied widely, ranging from 2 months to 20 years. Although the great toes are most commonly affected, other digits may also be involved. Patients may present with psoriatic skin lesions; however, these are not consistently observed.^1^ The key imaging feature of POPP is the involvement of the nail bed and phalanges in the absence of adjacent joint inflammation. Radiographically, whereas DIP PsA typically presents with synovitis, periarticular bone erosions, joint space narrowing, and destruction centered on the DIP joint, POPP is more often characterized by soft tissue prominence (dactylitis), periosteal new bone formation along the distal phalanx, and enthesopathic changes. Intra- and peri-articular erosions may also occur in cases with a long-term duration of symptoms.^1^ MRI is the preferred imaging modality in these patients due to its ability to detect bone marrow changes and provide better visualization of the nail bed. A characteristic finding is the thickening and contrast enhancement of the subungual tissues.^9^ A diagnostic algorithm is provided to summarize the key clinical and imaging features of POPP (Fig 6).

Treating POPP can be challenging, as there is no established standard of care. Among disease-modifying antirheumatic drugs, methotrexate is the most used, although treatment responses are variable.^1^^,^^2^ Case reports have shown that tumor necrosis factor inhibitors are more effective than disease-modifying antirheumatic drugs.^1^^,^^5^^,^^6^ Among the patients treated with anti-tumor necrosis factor agents, onychodystrophy has been reported as the symptom showing the least improvement. More recently, cases of refractory POPP have been successfully treated with secukinumab^1^ and guselkumab.^10^ Since ixekizumab has also demonstrated strong efficacy in both nail psoriasis and PsA in randomized trials,^11^^,^^12^ we opted to prescribe this therapy to our patient following a partial response to adalimumab. POPP is a rare and challenging condition with an uncertain pathogenesis and no established treatment guidelines. Our case highlights that interleukin-17 inhibitors, such as ixekizumab, may represent an effective therapeutic option for POPP. Further studies are needed to better define optimal management strategies for this uncommon disorder.

## Conflicts of interest

None disclosed.

## References

[1] Sakellariou G.T., Tsifountoudis I., Vounotrypidis P. (2021). Psoriatic onycho-pachydermo periostitis (POPP): a case report treated successfully with IL-17 blockade and a literature review on characteristics, pathogenesis, and treatment. Clin Rheumatol.

[2] Bauzá A., Redondo P., Aquerreta D. (2000). Psoriatic onycho-pachydermo periostitis: treatment with methotrexate. Br J Dermatol.

[3] Fournié B., Viraben R., Durroux R., Lassoued S., Gay R., Fournié A. (1989). [Psoriatic onycho-pachydermo-periostitis of the big toe. Anatomo-clinical study and physiopathogenic approach apropos of 4 cases]. Rev Rhum Mal Osteoartic.

[4] Boisseau-Garsaud A.M., Beylot-Barry M., Doutre M.S., Beylot C., Baran R. (1996). Psoriatic onycho-pachydermo-periostitis. A variant of psoriatic distal interphalangeal arthritis?. Arch Dermatol.

[5] Dans M., Hivnor C., Van Voorhees A.S. (2005). Psoriatic onychopachydermoperiostitis: improvement with etanercept. Br J Dermatol.

[6] Bongartz T., Härle P., Friedrich S. (2005). Successful treatment of psoriatic onycho-pachydermo periostitis (POPP) with adalimumab. Arthritis Rheum.

[7] Tsubota A., Abe R., Shibaki A. (2005). Clinical images: drumstick-like fingers indicating psoriatic onycho-pachydermo periostitis. Arthritis Rheum.

[8] Fietta P., Manganelli P. (2005). Childhood onset of psoriatic onycho-pachydermo-periostitis(POPP). J Eur Acad Dermatol Venereol.

[9] Hesni S., Khodatars D., Rees R., Khanna M., Walker M. (2022). Psoriatic onycho-pachydermo-periostitis. J Psoriasis Psoriatic Arthritis.

[10] Brunasso A.M.G., Sola S., Massone C. (2021). Recalcitrant psoriatic onycho-pachydermo-periostitis successfully treated with guselkumab. Clin Exp Dermatol.

[11] Mease P.J., van der Heijde D., Ritchlin C.T., SPIRIT-P1 Study Group (2017). Ixekizumab, an interleukin-17A specific monoclonal antibody, for the treatment of biologic-naive patients with active psoriatic arthritis: results from the 24-week randomised, double-blind, placebo-controlled and active (adalimumab)-controlled period of the phase III trial SPIRIT-P1. Ann Rheum Dis.

[12] van de Kerkhof P., Guenther L., Gottlieb A.B. (2017). Ixekizumab treatment improves fingernail psoriasis in patients with moderate-to-severe psoriasis: results from the randomized, controlled and open-label phases of UNCOVER-3. J Eur Acad Dermatol Venereol.

